# Prediction of Recurrent Atrial Tachyarrhythmia After Receiving Atrial Flutter Ablation in Patients With Prior Cardiac Surgery for Valvular Heart Disease

**DOI:** 10.3389/fcvm.2021.741377

**Published:** 2021-09-23

**Authors:** Ching-Yao Chou, Fa-Po Chung, Hung-Yu Chang, Yenn-Jiang Lin, Li-Wei Lo, Yu-Feng Hu, Tze-Fan Chao, Jo-Nan Liao, Ta-Chuan Tuan, Chin-Yu Lin, Ting-Yung Chang, Chih-Min Liu, Cheng-I Wu, Sung-Hao Huang, Chun-Chao Chen, Wen-Han Cheng, Shin-Huei Liu, Isaiah Carlos Lugtu, Ankit Jain, An-Ning Feng, Shih-Lin Chang, Shih-Ann Chen

**Affiliations:** ^1^Heart Rhythm Center and Division of Cardiology, Department of Medicine, Taipei Veterans General Hospital, Taipei, Taiwan; ^2^Division of Cardiology, Medical Center, Shin Kong Wu Ho Su Memorial Hospital, Taipei, Taiwan; ^3^Faculty of Medicine, School of Medicine, National Yang-Ming University, Taipei, Taiwan; ^4^Division of Cardiology, Heart Center, Cheng Hsin General Hospital, Taipei, Taiwan; ^5^Department of Medicine, National Yang-Ming University Hospital, Yilan, Taiwan; ^6^Heart Institute, Chinese General Hospital and Medical Center, Manila, Philippines; ^7^Vardhman Mahavir Medical College and Safdarjung Hospital, New Delhi, India; ^8^Cardiovascular Center, Taichung Veterans General Hospital, Taichung, Taiwan

**Keywords:** atrial flutter, valvular heart disease, ablation, atrial fibrillation, heart surgery

## Abstract

**Background:** Surgical scars cause an intra-atrial conduction delay and anatomical obstacles that facilitate the perpetuation of atrial flutter (AFL). This study aimed to investigate the outcome and predictor of recurrent atrial tachyarrhythmia after catheter ablation in patients with prior cardiac surgery for valvular heart disease (VHD) who presented with AFL.

**Methods:** Seventy-two patients with prior cardiac surgery for VHD who underwent AFL ablation were included. The patients were categorized into a typical AFL group (*n* = 45) and an atypical AFL group (*n* = 27). The endpoint was the recurrence of atrial tachyarrhythmia during follow-up. A multivariate analysis was performed to determine the predictor of recurrence.

**Results:** No significant difference was found in the recurrence rate of atrial tachyarrhythmia between the two groups. Patients with concomitant atrial fibrillation (AF) had a higher recurrence of typical AFL compared with those without AF (13 vs. 0%, *P* = 0.012). In subgroup analysis, typical AFL patients with concomitant AF had a higher incidence of recurrent atrial tachyarrhythmia than those without it (53 vs. 14%, *P* = 0.006). Regarding patients without AF, the typical AFL group had a lower recurrence rate of atrial tachyarrhythmia than the atypical AFL group (14 vs. 40%, *P* = 0.043). Multivariate analysis showed that chronic kidney disease (CKD) and left atrial diameter (LAD) were independent predictors of recurrence.

**Conclusions:** In our study cohort, concomitant AF was associated with recurrence of atrial tachyarrhythmia. CKD and LAD independently predicted recurrence after AFL ablation in patients who have undergone cardiac surgery for VHD.

## Introduction

In patients with prior cardiac surgery, atrial flutter (AFL) may develop with the critical isthmus located at the region bounded by surgical scar and the anatomical structure of the right or left atrium (1–5). Radiofrequency (RF) catheter ablation could be considered as the first-line therapy for atypical AFL when compared with antiarrhythmic drugs in patients with cardiac surgery history (6). It had been demonstrated that atypical AFL after surgery for congenital heart disease (CHD) could be successfully ablated in 50–90% of circuits with traditional entrainment or a three-dimensional (3D) mapping system (1–4, 7, 8). As for acquired heart disease, Aktas et al. had compared the ablation outcome of AFL between patients with and without prior cardiac surgery. The patients with prior cardiac surgery were likely to have a lower freedom from AFL and atrial fibrillation (AF) after AFL ablation than those without. The type of cardiac surgery was not emphasized in this study (9). Nabar et al. demonstrated that the success rate of ablation of atrial arrhythmia after open heart surgery (included CHD and valvular surgery) is ≥90%. The mean following period was relatively short term (12 months) (10). Enriquez et al. reported that macro-reentry was the predominant mechanism of atrial tachycardia (AT) in patients with mitral valve (MV) surgery. Repeated procedure could achieve a favorable outcome (11).

Our present study aimed to analyze the long-term outcomes after AFL catheter ablation in patients with prior cardiac surgery for valvular heart disease (VHD), including mitral, tricuspid, and aortic valve surgery and concomitant coronary artery bypass surgery (CABG). The type of flutter, impact of AF, and electrophysiologic mechanism were analyzed. Moreover, the predictors of recurrent atrial tachyarrhythmia for this patient group were determined.

## Methods

### Study Design and Patient Selection

This is a retrospective study that enrolled patients with a history of surgical intervention for VHD who underwent AFL catheter ablation. Two high-volume institutions in Taiwan were included in this study, namely, Taipei Veterans General Hospital and Cheng Hsin General Hospital. Eighty-two patients with a history of surgical intervention for VHD were included, all of whom received AFL catheter ablation between July 2008 and April 2019. Surgical intervention for VHD was defined as mitral valve, tricuspid valve, and aortic valve replacement or repair. Patients who received a concomitant CABG were also included. Clinical AF was defined as the diagnosis of AF before AFL catheter ablation. Concomitant AF ablation was not performed due to absence of clinical symptoms. The exclusion criteria included patients with AF who received pulmonary vein isolation (PVI) (*n* = 5), patients with focal AT who received catheter ablation (*n* = 3), and patients who received an incomplete procedure due to complications or unstable hemodynamics (*n* = 2). Finally, 72 patients were enrolled in the study. The patients were categorized into two groups according to the type of AFL catheter ablation they received: the typical AFL group (patients with typical AFL only; *n* = 45) and the atypical AFL group (patients who had an atypical AFL for catheter ablation, with or without concomitant typical AFL; *n* = 27). Baseline and electrophysiological characteristics were collected and analyzed.

This retrospective cohort study obtained ethical approval from the institutional review board of the Taipei Veterans General Hospital and was conducted in full compliance with national ethical and regulatory guidelines (11).

### Electrophysiological Study and Mapping

On surface 12-lead electrocardiography, a typical or atypical AFL could be identified by the flutter wave (12). Detailed electrophysiologic study and mapping has been described in our previous studies (13–15). Three-dimensional electroanatomical mapping systems were used in 26 (96.3%) of 27 patients in the atypical AFL group and 4 (8.9%) of 45 patients in the typical AFL group. The EnSite mapping system (St. Jude Medical, Minneapolis, MN, USA), Carto mapping system (Biosense-Webster Inc., Diamond Bar, CA, USA), Rhythmia (Boston Scientific Corporation, Middlesex County, MA, USA), and ablation catheters were used at the discretion of the treating electrophysiologist. The AFL circuit was confirmed using electroanatomical mapping and entrainment maneuvers.

### AFL Catheter Ablation

#### Typical AFL

Typical flutter was defined as common cavotricuspid isthmus (CTI)-dependent flutter including both counterclockwise (common) and clockwise (reverse common) variants, with the circuit originally described as a broad active wavefront rotating around the tricuspid annulus (16). With the traditional catheter mapping, concealed entrainment from the tricuspid isthmus was performed to confirm or exclude CTI-dependent flutter. A return cycle length after transient entrainment is equal to baseline cycle length (<20 ms difference) when pacing the flutter isthmus, right atrial roof, and anterior and septal right atrial walls (17). With the 3D mapping system, activation mapping should reveal the propagation around the tricuspid annulus. Ablation was performed by creating a linear lesion from the tricuspid annulus to the inferior vena cava. RF current was delivered through an 8-mm non-irrigated catheter using a temperature control mode with a maximal temperature of 60°C or 4-mm open-irrigated catheter using a power control mode with a maximal power of 30–40 W. Successful lesion formation was defined as a 90% reduction in electrogram amplitude. The endpoint of ablation was the bidirectional block across the line of ablation which was confirmed by differential pacing.

#### Atypical AFL

Atypical AFL was defined as the existence of non-CTI-dependent, macro-reentry circuit, which is common in the setting of prior atrial surgical scar. With the traditional catheter mapping, entrainment maneuver was performed at multiple sites and postpacing interval (PPI) not exceeding the cycle length by more than 20 ms was considered to be part of the circuit (18). With the 3D mapping system, activation mapping was used to reveal the flutter circuit. For right AFL, RF energy was delivered at the critical isthmus connecting to the anatomical obstacles (tricuspid annulus, inferior vena cava, or superior vena cava) until a 90% reduction in local electrogram amplitude was achieved and bidirectional block was observed. For left AFL, a transseptal puncture was performed with fluoroscopy guidance, and the activated clotting time was kept at ≥300 s with intravenous unfractionated heparin. RF energy was delivered to the critical isthmus connecting to the anatomical obstacles. In patients with scar-related AFL, the ablation strategy was to ablate the clinical AFL and any other potential flutter channel that could be identified. The endpoint of ablation was determined on the basis of AFL termination or interruption during ablation and negative inducibility of clinical AFL by programmed extra stimuli from the CS catheter with intravenous isoproterenol (1–5 μg/min) infused to achieve at least a 20% heart rate increment (19). The subsequent AFLs occurred owing to the interruption of the initial AFL circuit during ablation or induced by programmed stimulation after elimination of initial AFL.

### Follow-Up

After discharge following the index ablation procedure, the patients were followed up at 2 weeks and then regularly every 1–3 months at our cardiology outpatient clinic. Antiarrhythmic medicines were prescribed for 4–8 weeks after the procedure to prevent early recurrence of AF/AFL within 3 months. The blanking period was defined as within 3 months after ablation (20). Follow-up with 24-h Holter monitoring or 1-week cardiac event monitoring was performed 3 months after the ablation procedure and at any time if the patients experienced symptoms suggestive of tachyarrhythmia. Long-term efficacy was assessed based on resting surface 12-lead electrocardiograms (ECG), 24-h Holter monitoring records, and/or 1-week cardiac event monitoring records. Any atrial tachyarrhythmia such as AF, AFL, and AT lasting ≥30 s was defined as a recurrence. The follow-up period was up to September 30, 2019.

### Statistical Analysis

Continuous variables are expressed as mean ± standard deviation (SD). Categorical variables were compared using the chi-square test. Hazard ratio was calculated using univariate and multivariate logistic regression analyses with a stepwise analysis and confirmation. In the multivariate regression analysis, factors with *P* < 0.1 in the univariate analysis were selected for adjustment. The variables entered in the multivariate logistic regression analysis included chronic kidney disease (CKD) and left atrial diameter (LAD). A two-sided *P* < 0.05 was considered significant for all statistical determinations. All analyses were performed using IBM Statistical Product and Service Solutions (SPSS) Version 20.

## Results

### Patient Characteristics and AFL Catheter Ablation

**Figure 1:**
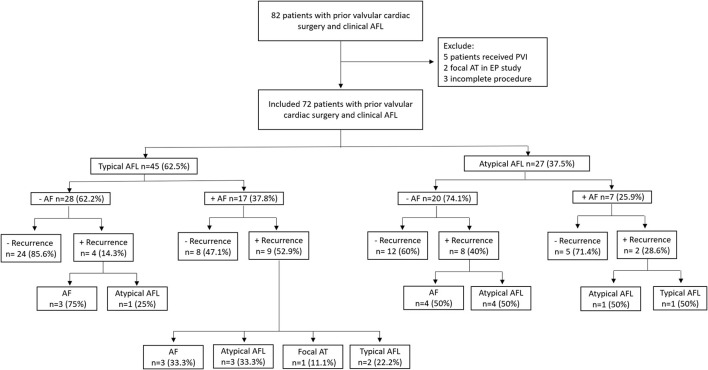
Flowsheet of the inclusion, classification of subgroups, recurrence number, and patterns in our study cohort. AFL, atrial flutter; AF, atrial fibrillation; AT, atrial tachycardia; PVI, pulmonary vein isolation; EP, electrophysiology.

The baseline characteristics are shown in Table 1. The flowsheet of the inclusion, exclusion, classification of subgroups, recurrence number, and patterns is provided in Figure 1. There were 45 patients in the typical AFL group and 27 patients in the atypical AFL group. No significant differences in baseline characteristics were found between these two groups, except for the higher incidence of hyperlipidemia in the atypical AFL group.

**Table 1:** Baseline characteristics of the study groups.

	**Study population** **(*n* = 72)**	**Typical AFL group** **(*n* = 45)**	**Atypical AFL group** **(*n* = 27)**	*P*-value^*^
Age (years)	59.9 ± 12.1	60.0 ± 11.7	59.8 ± 13.1	0.941
Male sex	38/72 (52.8%)	25/45 (56.5%)	13/27 (46.2%)	0.542
Hypertension	38/72 (52.8%)	22/45 (50.0%)	16/27 (57.7%)	0.393
Diabetes mellitus	13/72 (19.1%)	7/45 (17.4%)	6/27 (19.2%)	0.476
Chronic kidney disease	20/72 (27.8%)	12/45 (26.1%)	8/27 (30.8%)	0.786
Hyperlipidemia	19/72 (26.4%)	8/45 (17.4%)	11/27 (42.3%)	0.007
Congestive heart failure	21/72 (29.2%)	16/45 (34.8%)	5/27 (19.2%)	0.124
Coronary artery disease	17/72 (23.6%)	9/45 (17.4%)	8/27 (30.8%)	0.352
Atrial fibrillation	23/72 (31.9%)	17/45 (37.0%)	7/27 (23.1%)	0.302
Antiarrhythmic drug	39/72 (54.2%)	26/45 (57.8%)	13/27 (48.1%)	0.471
Left ventricular ejection fraction (%)	52.5 ± 12.4	51.2 ± 11.0	53.0 ± 8.8	0.476
Left ventricular dysfunction	16/72 (22.2%)	12/45 (26.1%)	4/27 (15.4%)	0.242
Left atrial enlargement	65/72 (90.3%)	42/45 (93.5%)	23/27 (84.6%)	0.259
Left atrial diameter (mm)	46.3 ± 7.5	47.4 ± 8.1	44.4 ± 6.2	0.107
Right atrial enlargement	14/72 (19.4%)	10/45 (21.7%)	4/27 (15.4%)	0.442
Multiple valve surgery	32/72 (44.4%)	20/45 (43.5%)	12/27 (46.2%)	1.000
Coronary bypass surgery	10/72 (13.9%)	6/45 (13.0%)	4/27 (15.4%)	0.860
Surgical AF ablation	9/72 (12.5%)	5/45 (11.1%)	4/27 (14.8%)	0.720

*AFL, atrial flutter; AF, atrial fibrillation*.

* *Comparison between the typical and atypical atrial flutter groups*.

The circuit number and the location of the isthmus of AFL in our study cohort are shown in Table 2. The mean number of reentrant circuits was 1.39 ± 0.72 in the total study population, while it was 2.04 ± 0.85 in the atypical AFL group. CTI-dependent AFL occurred in 17 patients in the atypical AFL group (17/27, 63.0%) and in 62 patients out of our total study cohort (62/72, 86.1%).

**Table 2:** The circuit number and the location of the isthmus of AFL in our study cohort.

	**Atypical AFL, *n* = 27**	**Typical AFL, *n* = 45**
Total circuit number	55	45
**RA**		
Circuit number	37/55 (67.3%)	45/45 (100%)
**Isthmus location**		
CTI	17/37 (45.9%)	45/45 (100%)
CT area^a^	7/37 (18.9%)	–
RA septal	6/37 (16.2%)	–
RA free wall	5/37 (13.5%)	–
SVC	2/37 (5.5%)	–
**LA**		
Circuit number	18/55 (32.7%)	–
**Isthmus location**		
LA septal	4/18 (22.2%)	–
LA anterior free wall	2/18 (11.1%)	–
LA posterior wall	1/18 (5.6%)	–
LA roof	3/18 (16.7%)	–
Mitral	5/18 (27.7%)	–
Pulmonary vein	3/18 (16.7%)	–
**Mean circuit number per patient**	2.04 ± 0.85	1

*AFL, atrial flutter; RA, right atrium; CTI, cavotricuspid isthmus; CT, crista terminalis; SVC, superior vena cava; LA, left atrium*.

a *The mechanism of AFL and the location of the isthmus were defined as in previous studies (21, 22)*.

**Figure 2:**
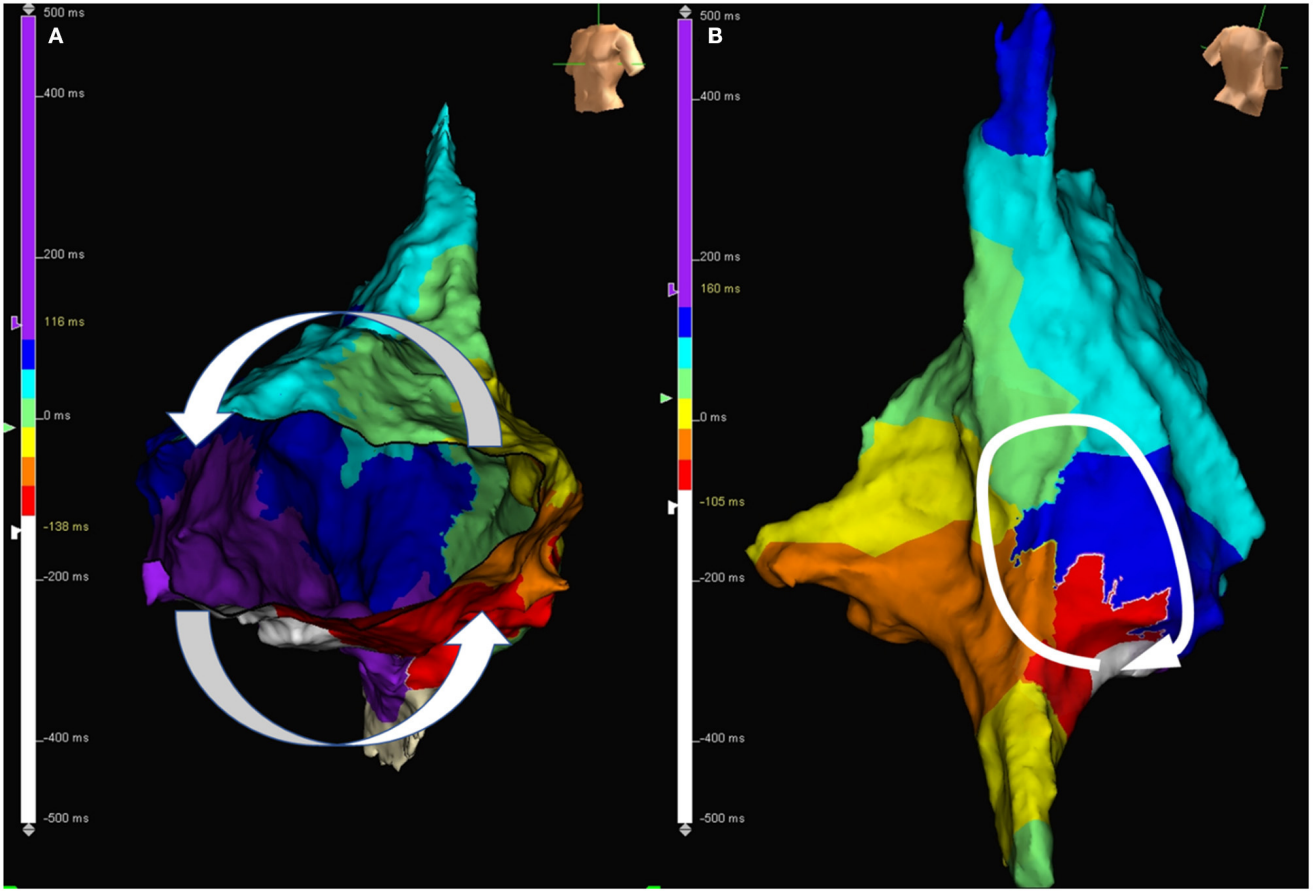
**(A)** Activation map during AFL in a patient with typical AFL. The activation wave front travels through cavotricuspid isthmus and goes around the tricuspid annulus. **(B)** Activation map during atypical atrial flutter in a patient with atypical AFL. The activation wave front goes around the surgical scar located at the right posterior free wall. AFL, atrial flutter.

**Figure 3:**
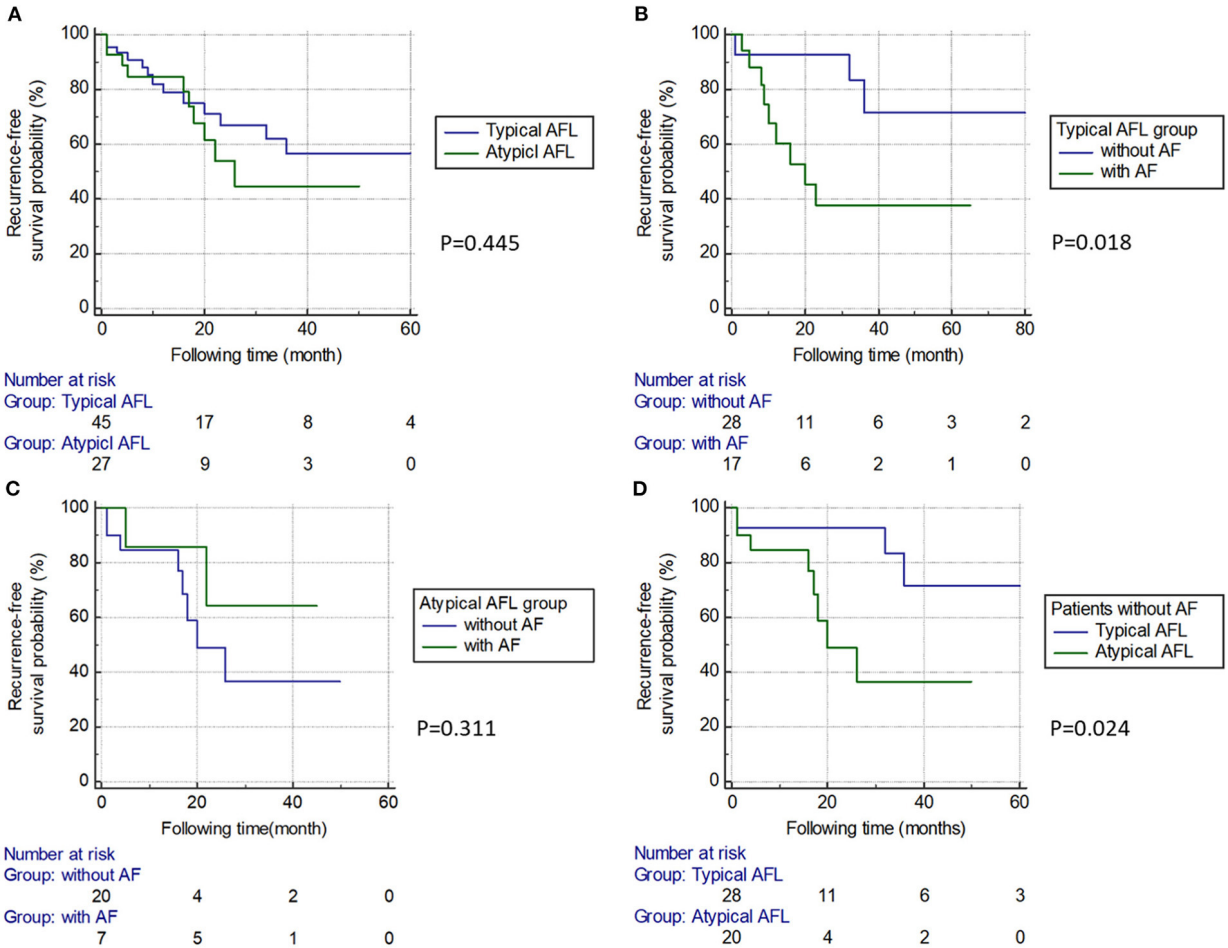
Kaplan–Meier survival curve between subgroups. **(A)** The typical and atypical AFL groups. There was no significant difference in the recurrence-free survival rate between the two groups. **(B)** Subgroups of patients with or without AF in the typical AFL group. Patients with AF had a higher recurrence rate than those without. **(C)** Subgroups of patients with or without AF in the atypical AFL group. There was no significant difference in the recurrence-free survival rate between the two groups. **(D)** In patients without AF, the atypical AFL group had a higher recurrence rate than the typical AFL group. AFL, atrial flutter; AF, atrial fibrillation.

An example of circuits in typical and atypical AFL demonstrated by the 3D mapping system is shown in Figure 2. In comparison of the typical and atypical AFL group, the mean follow-up period was 24.3 ± 26.0 and 18.0 ± 13.3 months, respectively (*P* = 0.18). No significant differences in the prevalence of clinical AF, recurrence rate of atrial tachyarrhythmia, and type of recurrent atrial tachyarrhythmias (including AFL, atypical AFL, AF, and focal AT) were found between the two groups (Table 3). The Kaplan–Meier analysis revealed no significant difference in the recurrence-free survival of atrial tachyarrhythmia between the typical and atypical AFL groups (Figure 3A).

**Table 3:** Ablation outcomes of the typical and atypical AFL groups.

	**Typical AFL group (*n* = 45)**	**Atypical AFL group (*n* = 27)**	***P*-value**
Following time (months)	24.3 ± 26.0	18.0 ± 13.3	0.180
CTI ablation	45/45 (100%)	17/27 (63.0%)	<0.001
Recurrence	13/45 (28.9%)	10/27 (37.0%)	0.473
Time to recurrence (months)	13.5 ± 11.4	13.0 ± 9.3	0.904
**Type of recurrent arrhythmia**			
Typical AFL	2/45 (4.4%)	1/27 (3.7%)	0.879
Atypical AFL	4/45 (8.9%)	5/27 (18.5%)	0.232
AF	6/45 (13.3%)	4/27 (14.8%)	0.860
Focal AT	1/45 (2.2%)	0/27 (0%)	0.435

### Impact of Clinical AF

As shown in Table 4, among 72 patients, 23 had concomitant AF (31.9%) before enrollment. Patients with concomitant AF had a trend toward a higher recurrence rate of atrial tachyarrhythmia compared to those without concomitant AF (*P* = 0.074). The recurrence rate of typical AFL was higher in patients with concomitant AF than those without concomitant AF (*P* = 0.012).

**Table 4:** Analysis of ablation outcomes among the different subgroups.

	**Patients** ** +AF** **(*n* = 24)**	**Patients** **–AF** **(*n* = 48)**	***P*-value**	**Typical** ** AFL +AF** **(*n* = 17)**	**Typical** ** AFL –AF** **(*n* = 28)**	***P*-value**	**Atypical** ** AFL +AF** ** (*n* = 7)**	**Atypical** ** AFL –AF** ** (*n* = 20)**	***P*-value**	**Typical** **AFL –AF** ** (*n* = 28)**	**Atypical** ** AFL –AF** ** (*n* = 20)**	***P*-value**
Recurrence	11/24 (45.8%)	12/48 (25.0%)	0.074	9/17 (52.9%)	4/28 (14.3%)	0.006	2/7 (28.6%)	8/20 (40.0%)	0.311	4/28 (14.3%)	8/20 (40.0%)	0.043
Time to recurrence (months)	12.1 ± 7.1	14.4 ± 12.8	0.592	11.8 ± 6.7	17.5 ± 19.1	0.597	13.5 ± 12.0	12.9 ± 9.6	0.938	17.5 ± 19.1	12.87 ± 9.53	0.673
**Type of recurrent arrhythmia**												
Typical AFL	3/24 (12.5%)	0/48 (0)	0.012	2/17 (11.9%)	0/28 (0)	0.063	1/7 (0)	0/20 (0)	0.085	0/28 (0)	0/20 (0)	–
Atypical AFL	4/24 (16.7%)	5/48 (10.4%)	0.450	3/17 (17.6%)	1/28 (3.6%)	0.108	1/7 (0%)	4/20 (20.0%)	0.738	1/28 (3.6%)	4/20 (20.0%)	0.066
AF	3/24 (12.5%)	7/48 (14.6%)	0.810	3/17 (17.6%)	3/28 (10.7%)	0.507	0/7 (10.7%)	4/20 (20.0%)	0.200	3/28 (10.7%)	4/20 (20.0%)	0.369
Focal AT	1/24 (4.2%)	0/48 (0)	0.154	1/17 (5.9%)	0/28 (0)	0.194	0/28 (0)	0/20 (0)	NA	0/28 (0)	0/20 (0)	–

In the typical AFL group, 17 out of 45 (37.8%) cases had a concomitant AF, and these patients had a significantly higher recurrence rate of atrial tachyarrhythmia than those without concomitant AF (*P* = 0.006; Table 4 and Figure 3B). In the atypical AFL group, we found no significant difference in the recurrence rate of atrial tachyarrhythmia between the patients with and without concomitant AF (*P* = 0.331; Table 4 and Figure 3C).

In patients without concomitant AF (*n* = 48), the atypical AFL group had a higher recurrence rate of atrial tachyarrhythmia than the typical AFL group (*P* = 0.043; Table 4 and Figure 3D).

### Impact of Cardiac Surgery

Among the 72 patients, 40 patients received single-valve surgery and 32 received multiple-valve surgery. Ten patients received concomitant on-pump CABG. The total number of valves treated by surgery in our study population was 110. The surgical detail is shown in Table 5.

**Table 5:** Surgical detail in our study population.

**VHD patients with AFL**, ***n*** **= 72**			
CABG	10/72 (13.9%)	On pump	10/10 (100%)
		Off pump	0/10 (0%)
Single valve	40/72 (55.6%)	MV	27/40 (67.5%)
		AV	12/40 (30.0%)
		TV	1/40 (2.5%)
Multiple valve	32/72 (44.4%)	MV+TV	20/32 (62.5%)
		MV+AV	5/32 (15.6%)
		AV+TV	2/32 (6.3%)
		MV+AV+TV	5/32 (15.6%)
**Total number of valves receiving surgery**, ***n*** **= 110**			
Annuloplasty	52/110 (47.3%)	MV	26/52 (50.0%)
		AV	1/52 (1.9%)
		TV	25/52 (48.1%)
Replacement	58/110 (52.7%)	MV	33/58 (56.9%)
		AV	23/58 (39.7%)
		TV	2/58 (3.4%)

*VHD, valvular heart disease; AFL, atrial flutter; CABG, coronary artery bypass graft; MV, mitral valve; TV, tricuspid valve; AV, aortic valve*.

In the comparison of the single-valve and multiple-valve groups, the occurrence rate of atypical AFL was the same (15/40 vs. 12/32, *P* = 1). In the single-valve surgery group, there was a trend toward an increased occurrence of atypical AFL in patients who underwent mitral valve (MV) surgery, when compared to aortic valve (AV) surgery (44.4 and 16.7%, *P* = 0.07, respectively). Only one patient received lone tricuspid valve (TV) surgery and developed atypical AFL.

LA flutter occurred in 10 patients in our study cohort (10/72, 13.9%), whereas the remaining 62 patients had RA flutter only (62/72, 86.1%). LA flutter was highly associated with prior MV surgery (9/10, 90%).

### Predictors of Arrhythmia Recurrence

Table 6 shows the results of the univariate and multivariate Cox regression analyses of each risk factor. In the univariate analysis, CKD and LAD were associated with the recurrence of atrial tachyarrhythmia (*P* = 0.017 and 0.029, respectively). In the multivariate analysis, CKD and LAD were both identified as independent predictors of recurrent atrial tachyarrhythmia (*P* = 0.02, hazard ratio 2.73 and *P* = 0.04, hazard ratio 1.06, respectively).

**Table 6:** Univariate and multivariate Cox regression analyses for each risk factor of recurrent atrial tachyarrhythmia.

	**Study population** **(*n* = 72)**	**Recurrence group (23/72, 32.4%)**	**Non-recurrence group (49/72, 67.6%)**	**Univariate** ***P*-value**	**Multivariate** ***P*-value**
Age (years)	59.9 ± 12.14	58.8 ± 12.1	60.4 ± 12.3	0.509	NA
Male sex	38/72 (52.78%)	14/23 (60.87%)	24/49 (48.98%)	0.408	NA
Hypertension	38/72 (52.78%)	13/23 (56.52%)	25/49 (51.02%)	0.428	NA
Diabetes mellitus	13/72 (18.06%)	6/23 (26.09%)	7/49 (14.29%)	0.200	NA
Chronic kidney disease	20/72 (27.78%)	10/23 (43.48%)	10/49 (20.41%)	0.017	0.026
Hyperlipidemia	19/72 (26.39%)	7/23 (30.43%)	12/49 (24.49%)	0.305	NA
Congestive heart failure	21/72 (29.17%)	7/23 (30.43%)	14/49 (28.57%)	0.940	NA
Coronary artery disease	17/72 (23.61%)	6/23 (26.09%)	11/49 (22.45%)	0.857	NA
Atrial fibrillation	23/72 (31.94%)	10/23 (43.48%)	13/49 (26.53%)	0.420	NA
Antiarrhythmic drug	39/72 (54.2%)	14/23 (60.9%)	25/49 (51.0%)	0.712	NA
Atypical AFL	26/72 (36.11%)	9/23 (39.13%)	14/49 (28.57%)	0.450	NA
Left ventricular ejection fraction (%)	52.5 ± 12.40	54.3 ± 10.5	51.6 ± 13.2	0.538	NA
Left ventricular dysfunction	16/72 (22.22%)	4/23 (17.39%)	12/49 (24.49%)	0.703	NA
Left atrial enlargement	65/72 (90.28%)	22/23 (95.65%)	43/49 (87.76%)	0.497	NA
Left atrial diameter (mm)	45.5 ± 7.8	48.2 ± 8.1	44.2 ± 7.4	0.029	0.044
Right atrial enlargement	14/72 (19.44%)	7/23 (30.43%)	7/49 (14.29%)	0.203	NA
Multiple valve surgery	32/72 (44.44%)	11/23 (47.83%)	21/49 (42.86%)	0.464	NA
Coronary bypass surgery	10/72 (13.89%)	2/23 (8.70%)	8/49 (16.33%)	0.801	NA
Surgical AF ablation	9/72 (12.5%)	2/23 (8.7%)	7/49 (14.3%)	0.836	NA

## Discussion

### Major Findings

We found no significant difference in the recurrence rate of atrial tachyarrhythmia between the typical and atypical AFL groups in our cohort. Patients with concomitant AF had a higher incidence of recurrent typical AFL than those without it. In the typical AFL group, patients with concomitant AF had a higher recurrence rate of atrial tachyarrhythmia than those without it. Among patients without concomitant AF, the atypical AFL group had a higher recurrence rate than the typical AFL group. LA flutter was less common than RA flutter and was highly associated with prior MV surgery. CKD and LAD were identified as predictors of recurrence of atrial tachyarrhythmia in patients who received AFL ablation after undergoing surgery for VHD.

### Recurrence of Atrial Tachyarrhythmia After AFL Ablation

Previous studies showed that typical AFL was the most common atrial arrhythmia and demonstrated that patients who had undergone cardiac surgery had higher recurrence rates of AFL (12 vs. 1%) and AF (28 vs. 16%) after AFL catheter ablation than those who had not undergone cardiac surgery (9, 10). Similarly, our present study showed that majority of the total study population (86%) had a CTI-dependent AFL. In the atypical AFL group, CTI-dependent AFL also occurred in 63.0% of the patients. The overall recurrence rate of atrial tachyarrhythmia in our study population was 31.9%. Surgical incisions, either left atriotomy or a trans-septal approach, provided a substrate for atrial arrhythmias, which may have contributed to the perpetuation of AFL (1–5, 23). A higher recurrence rate of atrial tachyarrhythmia after AFL ablation could be explained by the arrhythmogenic substrate in patients after cardiac surgery for VHD (24). As a result, these patients should be closely followed up even after successful elimination of the AFL during the ablation procedure.

### Impact of AF

Several studies have reported that successful AFL ablation decreased the AF recurrence in 50–75% of patients with a history of AF (25–33). One meta-analysis also demonstrated that the recurrence of AF was 52.7% in patients which a history of AF after a mean of 16 months following period (34). In our present study, the recurrence rate of AF was 12.5% after AFL ablation in patients with concomitant AF; however, the recurrence rate of AFL was 29.2% (12.5% typical AFL, 16.7% atypical AFL). In agreement with previous studies, AFL catheter ablation might be associated with decreased AF recurrence in our study cohort. The higher recurrence rate of AFL might be due to difference in arrhythmic substrate between patients with or without prior cardiac surgery. The underlying mechanism of the effects of AFL ablation on the reduction of AF recurrence rate remains unknown. We hypothesized that the presence of AFL could be a trigger to initiate other types of atrial tachyarrhythmias, and AFL could transform into AF over time. In contrast, in the typical AFL group, patients with concomitant AF had a higher recurrence rate of atrial tachyarrhythmia than those without it (52.9 vs. 14.3%). The presence of clinical AF may represent the diseased substrate and led to perpetuation of atrial tachyarrhythmia (35, 36).

Among patients without AF, a higher recurrence rate was found in the atypical AFL group than the typical AFL group in our study. This finding might be explained by atrial remodeling which is associated with a higher recurrence rate of atrial tachyarrhythmia. Patients with atypical AFL or multiple reentrant circuits might have a more complex and abnormal substrate than those with typical AFL only (14, 21).

### Impact of Cardiac Surgery

Cardiac surgery was associated with a high occurrence of atrial arrhythmias. AF and AFL have been reported in up to 15–40% of patients in the postoperative period following CABG, and a higher occurrence was found in valve surgery (37–50%) (37). RA incision and atriotomy were very common in valve surgery, and surgical scar contributed to more incidence of atypical AFL when compared with RA appendage cannulation which was performed in CABG. Since the location of the AFL circuit was highly associated with different surgical incision, RA flutter was much more common than LA flutter (38, 39). The occurrence of LA flutter was highly associated with LA surgical approach for MV surgery (23, 38–40). In our study cohort, LA flutter occurred in only 13% (10/72) of patients, and 90% (9/10) of these patients had MV surgery. This result was compatible with previous studies. Our study also demonstrated that the number of valves receiving surgery, concomitant CABG, and surgical AF ablation did not affect the occurrence of atypical AFL or recurrence after AFL ablation. Notably, in our cohort of single-valve surgery, there was a trend toward a higher occurrence of atypical AFL in MV surgery than AV surgery (*P* = 0.07).

### Predictor of Recurrence

CKD and LAD were predictors of recurrent atrial tachyarrhythmia in our present study. The correlation between CKD and AF has been demonstrated. The prevalence of AF in patients with CKD was found to be between 18 and 21.2%, and concomitant AF and CKD had a negative effect on prognosis (41–44). In patients with CKD without a preexisting AF, AF was detected in 7.7% of the patients after 5 years of follow-up (44). Several risk factors for developing atrial arrhythmias in patients with CKD have been reported: increased inflammation, lower serum calcium level, increased platelet count, and higher ferritin level (42, 45, 46). These factors could enhance the arrhythmogenic substrate contributing to the recurrence of atrial tachyarrhythmia after AFL ablation.

LA enlargement is involved in the mechanism of atrial arrhythmia. Several score systems including LA diameter have been used to evaluate the progression of AF (47). LA size was also an independent predictor of atrial tachyarrhythmia recurrence after AF ablation. One meta-analysis study showed three important predictors of recurrence after AF ablation: valvular AF, LAD longer than 50 mm, and recurrence within 30 days after ablation (48). LA volume seemed to be a more accurate predictor of recurrence than the type of AF (paroxysmal or persistent) (49–51). In agreement with previous evidence, our study suggested that LAD played an important role to predict the recurrence of atrial tachyarrhythmia in patients with cardiac surgery for VHD.

## Study Limitation

First, this was a retrospective study, and therefore, there were limitations inherent in retrospective data analysis and interpretation. Second, our study included 32% of the patients with preprocedural AF who received index AFL ablation without concomitant AF ablation. The reason why concomitant AF ablation was not done for those patients was because the clinical and predominant arrhythmia was AFL which caused the major symptom in our study population. Based on the decision of physicians, only AFL ablation was performed for those patients. Moreover, antiarrhythmic drugs were used in significant proportions of the included patients, and it is unclear whether we can exclude potentially associated AF. Third, follow-up with 24-h Holter monitoring or 1-week cardiac event monitoring was performed 3 months after the ablation procedure and whenever the patients experienced symptoms suggestive of tachyarrhythmia. The recurrence rate in our study may be underestimated because of asymptomatic arrhythmia during long-term follow-up. Finally, some patients received cardiac surgery in other hospitals not within the two centers in this study or had surgery a long time ago; hence, surgical records were not available for review for details. Although the AFL mechanism and isthmus location could be provided by the 3D mapping system, there was limitation to determine the relationship between the surgical technique and the flutter circuits in each case. Therefore, the classification of our study groups into typical and atypical AFL might be oversimplified for the complex nature of these patients.

## Conclusion

In our study cohort, we found no significant difference in the recurrence rate of atrial tachyarrhythmia between patients with typical and atypical AFL who had postsurgery VHD. In the typical AFL group, patients with AF had a higher recurrence rate of atrial tachyarrhythmia than those without AF. Among patients without concomitant AF, the atypical AFL group had a higher recurrence rate than the typical AFL group. LA flutter was less common than RA flutter and was highly associated with prior MV surgery. CKD and LAD were identified as independent predictors for recurrence of atrial tachyarrhythmia in patients who received AFL ablation with prior surgery for VHD. Close follow-up and monitoring of recurrent atrial tachyarrhythmia in this cohort are warranted.

## Data Availability Statement

The raw data supporting the conclusions of this article will be made available by the authors, without undue reservation.

## Ethics Statement

This retrospective cohort study obtained ethical approval from the institutional review board of the Taipei Veterans General Hospital. Written informed consent for participation was not required for this study in accordance with the national legislation and the institutional requirements.

## Author Contributions

C-YC: data collection and writing. H-YC, C-YL, T-YC, C-ML, C-IW, S-HH, C-CC, W-HC, S-HL, IL, and AJ: help for data collection. F-PC, Y-JL, L-WL, Y-FH, T-FC, J-NL, T-CT, A-NF, and S-AC: supervision and manuscript correction. S-LC: writing review and edition. All authors contributed to the article and approved the submitted version.

## Funding

This work was supported by Taipei Veterans General Hospital grants (V108C-055, V108C-032, C17-095, C19-027, V107C-041, V106C-056, VGHUST107-G1-7-1, MOST 107-2314-B-010-061-MY2, MOST 106-2314-B-010-046-MY3, MOST 106-2314-B-010-035-MY3, MOST 105-2314-B-075-036, MOST104-2314-B-075-0, and MOST104-2314-B-075-024-MY3) and the SZU-YUAN Research Foundation of Internal Medicine.

## Conflict of Interest

The authors declare that the research was conducted in the absence of any commercial or financial relationships that could be construed as a potential conflict of interest.

## Publisher's Note

All claims expressed in this article are solely those of the authors and do not necessarily represent those of their affiliated organizations, or those of the publisher, the editors and the reviewers. Any product that may be evaluated in this article, or claim that may be made by its manufacturer, is not guaranteed or endorsed by the publisher.
